# Feasibility, and barriers to use escitalopram in functional gastrointestinal disorders

**DOI:** 10.3389/fphar.2023.1131354

**Published:** 2023-05-22

**Authors:** Saad S. Alkhowaiter, Amani H. Alshahrani, Hala F. Almarzouqi, Gadah K. Alonazi, Tariq M. Alhawassi, Maha M. AlRasheed

**Affiliations:** ^1^ Department of Medicine- Gastroenterology, College of Medicine, King Saud University, Riyadh, Saudi Arabia; ^2^ Department of Clinical Pharmacy, College of Pharmacy, King Saud University, Riyadh, Saudi Arabia

**Keywords:** irritable bowel syndrome, functional heartburn, globus sensation, escitalopram, functional gastrointestinal

## Abstract

**Background and study aims:** The feasibility and barriars of escitalopram use in patients with functional gastrointestinal disorders (FGIDs) are still debated. We aimed to evaluate the feasibility, safety and efficacy and barriars of escitalopram use in managing FGIDs in the Saudi population.

**Patients and Methods:** We included 51 patients who received escitalopram for irritable bowel syndrome (n = 26), functional heartburn (n = 10), globus sensation (n = 10) or combined disorders (n = 5). We used an irritable bowel syndrome-severity scoring system IBS-SSS), GerdQ questionnaire and Glasgow Edinburg Throat Scale (GETS) to assess disease severity change before and after treatment.

**Results:** The median age was 33 years (25th- 75th percentiles: 29–47), and 26 (50.98%) were males. Forty-one patients experienced side effects (80.39%), but most side effects were mild. The most common side effects were drowsiness/fatigue/dizziness (54.9%), xerostomia (23.53%), nausea/vomiting (21.57%) and weight gain (17.65%). IBS-SSS was 375 (255–430) and 90 (58–205) before and after treatment, respectively (*p* < 0.001). GerdQ score was 12 (10–13) before treatment and 7 (6–10) after treatment (*p* = 0.001). GETS score before treatment was 32.5 (21–46) and after treatment became 22 (13–31) (*p* = 0.002). Thirty-five patients refused to take the medications, and seven patients discontinued the medication. Possible causes of the poor compliance were fear of the medications and not being convinced of taking psychiatric medications for functional disorders (n = 15).

**Conclusion:** Escitalopram could be a safe and effective treatment for functional gastrointestinal disorders. Targeting and managing factors leading to poor compliance could further improve the treatment outcome.

## Introduction

Functional gastrointestinal disorders (FGIDs) are highly prevalent, and a study showed that 40% of people worldwide suffer from FGIDs (Sperber et al., 2021). Nevertheless, FGIDs deeply affect the quality of life and increase healthcare-related costs (Drossman, 2016). Both physiological and psychological factors may contribute to gastrointestinal symptoms. Moreover, psychosocial factors can influence gastrointestinal (GI) tract physiological function via the brain-gut axis and lead to a greater response in FGID patients when compared to healthy subjects (Drossman, 2016). Neuromodulators could act peripherally or centrally to modulate the course of FGIDs (Törnblom and Drossman, 2018).

The use of selective serotonin reuptake inhibitors (SSRIs) in FGIDs has been evaluated before; however, their safety and efficacy are still debated. British Society of Gastroenterology guidelines on the management of irritable bowel syndrome (IBS) state that tricyclic antidepressants (TCA) and SRRIs can be used as second-line therapy for IBS (Vasant et al., 2021). According to the guidelines, it is reasonable to consider using TCAs second line to treat global symptoms or abdominal pain or SSRIs second line to treat global symptoms, or if there is coexistent anxiety; therefore, both agents can be used. Despite that, SSRIs were found to be effective in the management of selective GI motility disorders (Viazis et al., 2011). Ladabaum and colleagues found no benefit of escitalopram over placebo in managing IBS (Ladabaum et al., 2010). Manolakis and coworkers found that SSRIs exert a variable effect on esophageal motility and may induce globus sensation (GS) (Manolakis et al., 2019).

Currently, the evidence supporting the use of escitalopram in managing functional GI disorders is still weak (Vasant et al., 2021). Moreover, the Saudi population is underrepresented in the literature. Several barriers may prevent the wide use of escitalopram in managing functional GI disorders in the Saudi population. The objective of this study was to evaluate the feasibility and barriers to using escitalopram in managing IBS, functional heartburn, and GS in the Saudi population. Additionally, we assessed the safety and efficacy of escitalopram for functional GI disorders in the Saudi population.

## Patients and methods

### Design and patients

We conducted an ambispective study to assess the feasibility of using escitalopram for FGIDs in the Saudi population. Our inclusion criteria were adult patients (18 years and above) with FGIDs, including functional heartburn, IBS, and GS. We identified 173 patients prescribed escitalopram from the GI clinic in 2020. Patients were recruited if they met Rome IV criteria; all patients had symptoms for more than 6 months before enrollment (Lacy and Patel, 2017).

We assessed their baseline symptoms retrospectively using physician notes, electronic health records, and data collection sheets. All patients had a prospective follow-up by phone calls between January and February 2021 to assess the improvement of symptoms using validated scales and physician notes. We excluded 14 patients (post-treatment exclusion) for meeting the following criteria: patients who used escitalopram for other medical conditions, pregnant or nursing women, gastrointestinal or systemic illnesses that could affect gastrointestinal motility, the use of medications that may alter motility or interact with the study medications, patients diagnosed with inflammatory bowel disease, and psychiatric or psychological problem. Twelve patients did not want to answer our questionnaire and withdrew from the study. Additionally, we found that seven patients stopped the drug, and 35 patients did not take the medication at all.

**FIGURE 1:**
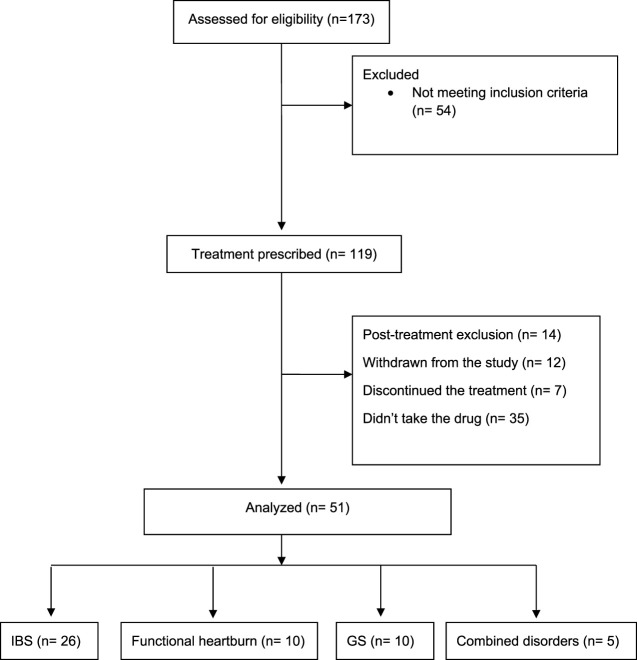
Study flow diagram. GS, globus sensation; IBS, irritable bowel syndrome.

Fifty-one patients were included in the final analysis (IBS = 26, Functional heartburn = 10, GS = 10, combined = 5). The study flow diagram is presented in Figure 1. All patients received SSRIs as second-line therapy. In patients with IBS, all received 1^st^ line medications according to their symptoms, such as loperamide, anti-spasmodic, or laxatives, in addition, to counseling about the disease. Patients with heartburn received proton pump inhibitors, with no adequate response, including counseling in the clinic. Patients with globus sensation received adequate counseling with lifestyle modifications. In the clinic and before starting SRRIs, we explained to the patients that SSRI is a neuromodulator that would help to improve gut-brain axis dysfunction. It was explained that these medications are used for depression as well, hence named antidepressants. The patients were informed about potential side effects before starting the medications.

The study was approved by the Local Ethical Committee, and the patients’ consents were obtained prior to enrollment.

### Definitions

FGDIs were diagnosed based on Rome IV criteria (Yamasaki et al., 2017). Functional heartburn was diagnosed in the presence of heartburn with negative cardiac workup, normal upper GI endoscopy, and normal ambulatory PH study. IBS was diagnosed with abdominal pain recurring at least 1 day per week for at least 3 months and associated with defecation, change in stool frequency, or appearance. GS was diagnosed with persistent or intermittent feelings of non-painful foreign body in the throat for 6 months.

## Instruments

### Irritable bowel syndrome severity scoring system

We assessed the severity of IBS symptoms using the irritable bowel syndrome-severity scoring system (IBS-SSS). The severity score questionnaire consisted of 8 questions with a maximum achievable score of 500. The score was interpreted as remission (<75), mild (from 75 to 174), moderate (from 175 to 300), and severe (above 300) (Francis et al., 1997).

### GerdQ questionnaire

The severity of heartburn symptoms was assessed using the GerdQ questionnaire. It assessed the positive predictors of gastroesophageal reflux disease (heartburn, regurgitation, sleep disturbance because of reflux symptoms, and the use of over-the-counter medications for heartburn) using a Likert scale (0–3). A reversed Likert scale was used to assess the negative predictors of GERS (epigastric pain and nausea). The GerdQ score ranged from 0 to 18. A score of 8 or more has a high probability of GERD (AlTassan et al., 2020).

### Glasgow Edinburg Throat Scale

The severity of globus symptoms was assessed using the Glasgow Edinburg Throat Scale (GETS) (Deary et al., 1995). The highest possible GETS score was 70, and we classified patients as asymptomatic (0–2), mildly symptomatic (3–8), symptomatic (9–20), and strongly symptomatic (more than 20).

### Side effects evaluation

Side effects of escitalopram were evaluated for all patients during a phone call asking the patients standardized questions about all possible side effects they could have experienced.

### Questionnaire validation

Two independent translators translated the questionnaires into Arabic and then back to English. Experts evaluated the initially translated questionnaires for clarity, simplicity, and the importance of questions. A pilot study was conducted on 12 subjects, and modifications from this study were integrated to constitute the final questionnaire.

### Statistical analysis

The normality distribution of the continuous variables was assessed using the Shapiro-Wilk test. Continuous data were presented as median (25th and 75th percentiles). Binary and ordinal data were presented as frequencies and percentages. Wilcoxon signed-rank test for matched pairs was used to compare continuous variables before and after treatment (GerQ and GETS scores). Baseline IBS score was compared between males and females using the Wilcoxon test. Ordinal data before and after treatment were compared using the Friedman test. A univariable mixed-effect model was used to identify variables affecting IBS-SSS change before and after treatment, and the β coefficient of the regression model was reported. Univariable logistic regression analysis with reporting the odds ratio was used to evaluate factors associated with treatment complications. Data were presented graphically with histograms, box plots, and Pareto charts. Data analysis was performed using Stata 16.1(Stata Corp- College Station- TX- United States) and Microsoft Excel (Microsoft, Redmond, WA, United States). Patients who were on escitalopram for functional GI disorders were evaluated for medication safety and efficacy (per protocol analysis). Additionally, patients who discontinued the medication and those who refused to take the medication were included with compliant patients for evaluation of non-adherence.

## Results

### Baseline socio-demographic data

The baseline data of the included 51 patients are presented in Table 1. The median age was 33 years (25th- 75th percentiles: 29–47). The most common diagnosis was IBS (n = 26%, 50.98%), followed by functional heartburn and GS (n = 10, 19.61%). Five patients had combined disorders (9.8%). The duration of treatment was 107 (60–165) days. Seven patients (33.33%) had treatment for less than 3 months, 22 patients (43.14%) had treatment between 3 and 6 months, and 12 patients (23.53%) had treatment for more than 6 months.

**TABLE 1:** The baseline socio-demographic data for patients with functional gastrointestinal disorders treated with escitalopram.

Variable	(n = 51)
Age (years) median (25th- 75th percentiles)	33 (29–47)
Weight (Kg) (25th- 75th percentiles)	70 (60–84)
Height (cm) (25th- 75th percentiles)	166 (158–173)
BMI (Kg/m2) (25th- 75th percentiles)	25.1 (21.6–29.4)
Male, n (%)	26 (50.98%)
Smoking, n (%)	
Yes	15 (29.41%)
No	32 (62.75%)
Previous smoker	4 (7.84%)
Employed, n (%)	34 (66.67%)
Educational level, n (%)	
High school and below	9 (17.65%)
Bachelor’s degree	33 (64.71%)
Diploma	2 (3.92%)
Master’s degree and above	7 (13.73%)
Married, n (%)	27 (52.94%)
Have children, n (%)	26 (50.98%)
Spicy food consumption, n (%)	12 (23.53%)
Coffee/tea drinking, n (%)	44 (86.27%)
Fast food consumption, n (%)	13 (25.49%)
Soda consumption, n (%)	9 (17.65%)
Exercise, n (%)	34 (66.67%)
Previous surgery, n (%)	33 (64.71%)
History of chronic disease, n (%)	13 (25.49%)
Concomitant medications, n (%)	
Proton pump inhibitors	12 (23.53%)
Anticholinergic	4 (7.84%)
Eucarbon	1 (1.96%)
Dopamine antagonist	1 (1.96%)
Combined	2 (3.92%)
Diagnosis, n (%)	
Inflammatory bowel disease (IBS)	26 (50.98%)
Functional heartburn	10 (19.61%)
Globus sensation	10 (19.61%)
IBS and functional heartburn	1 (1.96%)
IBS and globus sensation	2 (3.92%)
IBS, functional heartburn, globus sensation	1 (1.96%)
Functional heartburn and globus sensation	1 (1.96%)

### Escitalopram safety

Forty-one patients experienced side effects after using escitalopram (80.39%). Most of the side effects were mild and did not require drug discontinuation. The most common side effects were drowsiness/fatigue/dizziness (54.9%), xerostomia (23.53%), nausea/vomiting (21.57%), and weight gain (17.65%). (Table 2).

**TABLE 2:** Side effects reported after the use of escitalopram for functional gastrointestinal disorders.

	(n = 51) n (%)
Apathy, n (%)	3 (5.88%)
Xerostomia, n (%)	12 (23.53%)
Diaphoresis, n (%)	7 (13.73%)
Abnormal dreams, n (%)	2 (3.92%)
Flu like symptoms, n (%)	6 (11.76%)
Weight loss, n (%)	2 (3.92%)
Weight gain, n (%)	9 (17.65%)
Increase appetite, n (%)	2 (3.92%)
Decrease appetite, n (%)	1 (1.96%)
Headache, n (%)	5 (9.80%)
Nausea and vomiting, n (%)	11 (21.57%)
Numbness, n (%)	3 (5.88%)
Sexual disorders, n (%)	2 (3.92%)
Yawning, n (%)	10 (19.61%)
Diarrhea/Constipation, n (%)	6 (11.76%)
Palpitation, n (%)	1 (1.96%)
Abdominal bloating, n (%)	3 (5.88%)
Abdominal cramps, n (%)	1 (1.96%)
Drowsiness/Fatigue/Dizziness, n (%)	28 (54.90%)
Agitation/depression, n (%)	2 (3.92%)
Abdominal pain, n (%)	1 (1.96%)
Insomnia, n (%)	3 (5.88%)
Involuntary movement, n (%)	1 (1.96%)
Mood changes, n (%)	1 (1.96%)

We assessed the association between the socio-demographic variables and the occurrence of complications. There was no association between complications and the duration of treatment (OR: 0.99 (95% CI: 0.99–1); *p* = 0.16), the diagnosis (OR: 1.1 (0.56–2.18); *p* = 0.78) or the use of other medications (OR: 0.89 (0.60–1.32), *p* = 0.57). There was a tendency to report more complications in patients with lower educational levels, but it did not reach a significant value (OR: 0.5 (0.23–1.1); *p* = 0.07).

### Escitalopram efficacy

The efficacy of escitalopram was assessed with the change in the symptoms after treatment. Thirty-nine patients reported improvement (81.25%), five patients reported no improvement (10.42%), and four patients responded with “Maybe” (8.33%). The median degree of improvement was 70% (40–80), and the time to first feeling of improvement was 30 (14–30) days.

### Irritable bowel syndrome symptoms

Thirty patients with IBS were evaluated. Abdominal bloating was the most common symptom of IBS (n = 20). After treatment, one patient reported the disappearance of symptoms, 15 had improvement, 3 had the same sensation, and one had worsening symptoms. Before treatment, three patients had mild symptoms (10%), 4 had moderate (13.33%), 22 had severe (73.33%) symptoms, and 1 had remission (3.33%). After treatment, 6 had mild (20%), 8 had moderate (26.67%), 2 had severe (6.67%), and 14 had remission (46.67%) (*p* < 0.001).

**FIGURE 2:**
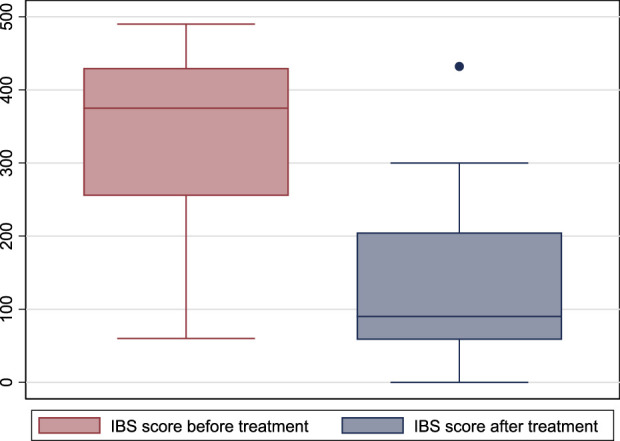
Box plot of irritable bowel syndrome-severity scoring system before and after treatment with escitalopram. (IBS: irritable bowel syndrome).

The IBS score before treatment did not differ between males and females [368 (25th- 75th percentiles: 238–430) vs. 385 (320–430) in males vs. females, respectively; *p* = 0.659]. IBS score before treatment was 375 (25th- 75th percentiles: 255–430) and was 90 (58–205) after treatment (*p* < 0.001). (Figure 2). The IBS-SSS significantly decreased after treatment, and older age and male gender were associated with a significant decrease in IBS-SSS. While the duration of treatment, concomitant medications, body mass index, and the associated chronic disease did not affect the score. (Table 3).

**TABLE 3:** Factors affecting the change in irritable bowel syndrome-severity scoring system.

	β (95% confidence interval)	*p*-value
Age	−3.52 (−5.73–−1.3)	0.002
Female	59.01 (3.51–114.51)	0.037
Body mass index (BMI)*	−1.72 (−6.66–3.23)	0.497
Associated chronic disease	0.49 (−55.75–56.73)	0.986
Duration of treatment	−20.22 (−59.46–19.02)	0.312
The use of other medications	−7.47 (−22.03–7.1)	0.315
Escitalopram	−209.97 (−260.46–−159.48)	<0.001

*BMI, was tested as a continuous variable.

### Functional heartburn symptoms

Thirteen patients with functional heartburn were evaluated. Eight patients complained of heartburn. After treatment, seven improved, and one had the same sensation. Six patients had regurgitation; five improved after treatment, and one stayed the same. The symptoms did not get worse in any patient.

**FIGURE 3:**
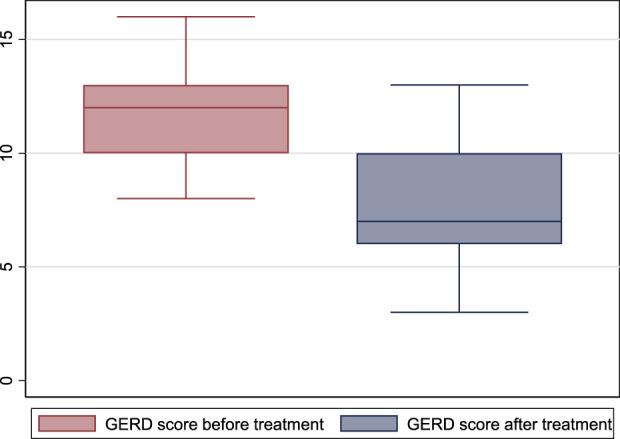
GerdQ score before and after treatment with escitalopram. (GERD: gastroesophageal reflux disease).

GerdQ score was 12 (10–13) before treatment and 7 (6–10) after treatment (*p* = 0.001) (Figure 3).

### Globus sensation symptoms

Fourteen patients with GS were evaluated. One patient recovered after treatment, nine improved, and one had the same degree of discomfort.

**FIGURE 4:**
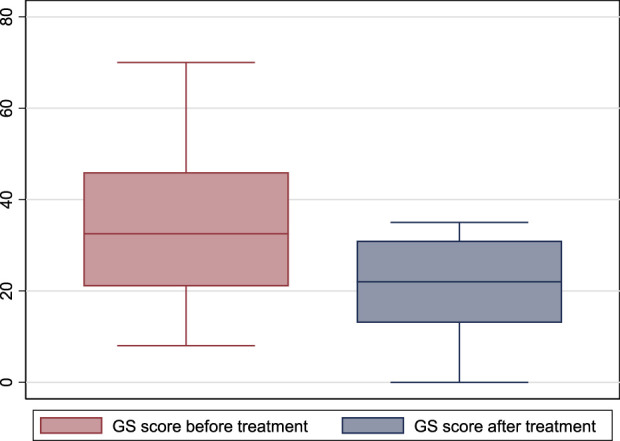
The global globus sensation score before and after treatment with escitalopram.(GS: global sensation).

One patient had mildly symptomatic GS before treatment, one had moderately symptomatic, and 12 had severely symptomatic GS. After treatment, one patient had no symptoms, one had mild symptoms, four had moderate symptoms, and eight had severe symptoms (*p* = 0.29). However, the GETS score before treatment was 32.5 (21–46), and after treatment became 22 (13–31) (*p* = 0.002). (Figure 4).

### Barriers against the use of escitalopram for FGIDs in the Saudi population

We identified seven patients who discontinued the drug within a few days of use. Reasons for drug discontinuation were fear of the medication (n = 2), not being convinced of taking psychiatric medications for FGIDs (n = 2), symptoms improved with other medicines (n = 1), symptoms did not improve after a few days of use (n = 1) and without an apparent reason (n = 1).

**FIGURE A1:**
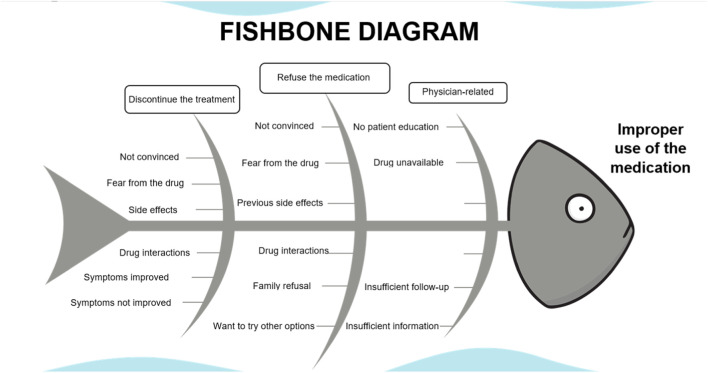
Fishbone chart for possible causes of treatment failure

**FIGURE A2:**
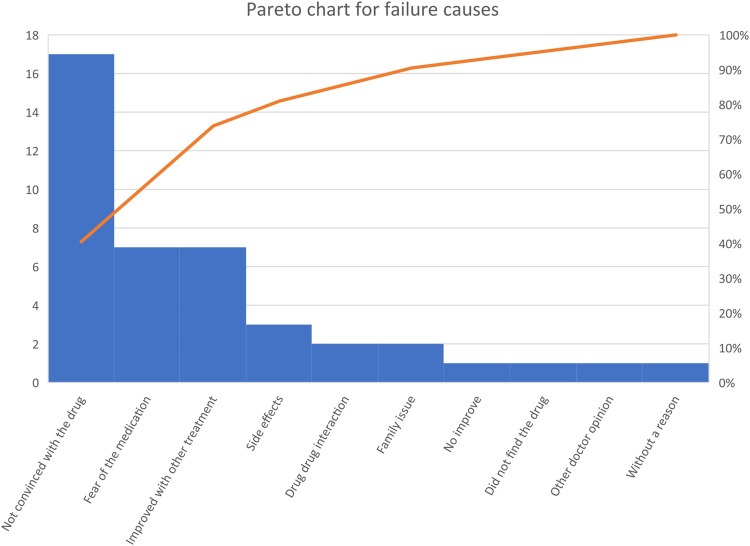
Pareto chart for causes of discontinuation or refusal to take the medication. “A Pareto chart is a bar graph in which the variables are plotted according to the relative frequency. The variables of the highest frequency are plotted from left to right. The orange-colored line presents the cumulative frequency”.

Thirty-five patients refused to take the medications. Reasons for refusal were fear of the medication (n = 5), not being convinced of taking psychiatric medications for functional GID (n = 15), side effects reported on previous use (n = 3), improvement with other medications or lifestyle change (n = 6), the patient did not find the medication (n = 1), fear from drug-drug interaction (n = 2), other doctor opinions against the use of the medication (n = 1), and family refusal (n = 2). (Figures A1, A2).

## Discussion

We performed a retrospective study to evaluate the feasibility and barriers of using escitalopram to manage FGIDs in the Saudi population. Fifty-one patients were on escitalopram for IBS, functional heartburn, and GS and were evaluated for medication safety and efficacy. Escitalopram safety was assessed with the frequency of side effects after treatment and efficacy with the change in patients’ symptoms and disease severity scores. Additionally, patients who discontinued the medication (n = 7) and those who refused to take the medication (n = 35) were added to evaluate non-adherence. We found 45% of the patients (42/93) were non-adherent with the prescription, and we identified possible causes of poor compliance.

### Escitalopram safety

We confirmed the results of several studies about the safety of escitalopram for patients with FGID (Thiwan and Drossman, 2006). We did not report serious side effects after use; the patients tolerated most symptoms. In several studies, tricyclic antidepressants relieved abdominal pain and diarrhea and improved slow colonic transit times. However, tricyclic antidepressants have numerous side effects compared to SSRIs (Talley et al., 2015; Törnblom and Drossman, 2018).

### Escitalopram efficacy

We reported improvement in the disease severity scores in all disease categories included. Results from the literature about the efficacy of SSIs in FGID are controversial. In our study, most patients reported the disappearance or improvement of their symptoms after treatment. Viazis and associates conducted a randomized placebo-controlled study on 252 patients to assess the effect of SSRIs on patients with hypersensitive esophagus. The study showed that 15 out of the 39 patients who received SSRIs (38.5%) and 24 out of 36 who received placebo (66.7%) continued to have reflux symptoms (*p* = 0.02) (Viazis et al., 2011). In a clinical trial on 40 IBS patients, Kuiken and coworkers found that fluoxetine significantly reduced the number of patients reporting significant abdominal pain; however, it did not affect rectal sensitivity (Kuiken et al., 2003).

In a larger trial, Broekaert and coworkers found that SSRIs did not affect esophageal motility but decreased chemical and mechanical sensitivity (Broekaert et al., 2006). Ladabaum and associates found no superiority for escitalopram over placebo for IBS (Ladabaum et al., 2010). Another study found a decrease in brain response to esophageal acid infusion with nortriptyline but without clinical significance (Forcelini et al., 2014). Talley and coworkers found that amitriptyline could benefit some patients with FGIDs, a finding that was not found with escitalopram (Talley et al., 2015).

### Barriers against the use of escitalopram

Even though much evidence has proven the beneficial role of psychotropic drugs such as antidepressants and anti-anxiolytic agents in the treatment FGIDs, as they act on the central nervous system and GI tract, their efficacies in GI symptoms do not rely on psychological symptom changes. Moreover, patients with FGIDs have concerns and negative perceptions about psychotropic medications (Xiong et al., 2018a).

Unfortunately, social stigma and misconceptions around psychiatric medications still exist, which could be a barrier against using a beneficial medication in a non-psychiatric disorder such as FGID. Besides, antidepressants take several weeks to demonstrate full effectiveness. On the other hand, adverse events occur more quickly, which can be a source of poor compliance or early dropout of medication. Like any drug, escitalopram can have various side effects, but these side effects may improve over time (Xiong et al., 2018b).

Several other reasons for poor compliance were identified (López-Torres et al., 2013). These include side effects, fear of intolerance, dependence or inappropriate treatment, and other reasons attributed to healthcare providers who erroneously tell the patient to discontinue treatment.

Non-adherence to SRRIs was evaluated before; however, the evaluation of non-adherence to these medications in patients with FGID was not performed before. We found that two main types of patients were non-adherence were those who were not convinced by the medication and those who feared to use it. SRRIs’ side effects were our cohort’s fourth cause of non-adherence, and lack of improvement was the seventh cause. Another reported cause of non-adherence was family issues, which could be more evident in our community, where family opinion may affect the decision to use specific medications. This factor requires societal efforts to change the social stigmata for using psychiatric drugs.

Antidepressants interact with different receptors, explaining both these drugs’ wanted and unwanted effects. In addition, tolerability is inseparably linked to patient compliance and, ultimately, to the overall success of treatment. Among new antidepressants, SSRIs show favorable overall tolerability and safety compared to other antidepressants. Cassell and colleagues showed that subject medication adherence correlated positively with perceived medication necessity and negatively concerned medication harm (Cassell et al., 2015).

Therefore, multifaceted interventions are mandatory for patients with FGIDs treated with SSRIs. These include proper education of the patients and their families regarding the nature of FGIDs available treatment options, the time it takes to see a response, early side effects and what to do about them, and the expected course of treatment (Chong et al., 2011). Discussing the risks and benefits before starting the medication could influence the treatment process, patient satisfaction, and medication adherence. Gradual starting and withdrawal may help to decrease unwanted side effects. Other interventions to improve compliance include using well-tolerated drugs with a simple administration regimen and family support.

### Study significance and limitations

This study highlighted the feasibility, safety, and efficacy of escitalopram for the management of FGIDs. We did not report serious side effects that warranted drug discontinuation or affected normal daily activity. Additionally, the disease severity scores have improved after using the medication. These results indicate the safety and efficacy of escitalopram in FGIDs. We identified the factors that could lead to poor compliance that should be addressed when designing future studies for escitalopram in FGIDs in the Saudi population. The study’s retrospective design and inherent referral and selection biases are major limitations. The study is limited by the small sample size, especially for patients in GS and the functional heartburn group. However, the study showed significant improvement in symptoms after treatment.

In conclusion, escitalopram could be a safe and effective treatment for functional gastrointestinal disorders. Targeting and managing factors leading to poor compliance could improve the treatment outcome. The confirmation of our findings in a larger randomized trial is recommended.

## Data Availability

The raw data supporting the conclusion of this article will be made available by the authors, without undue reservation.
